# Genome Sequences of Two *Salmonella enterica* Serovar Kentucky Isolates Recovered from Poultry Carcasses in the United States

**DOI:** 10.1128/genomeA.01289-16

**Published:** 2016-11-17

**Authors:** Bradd J. Haley, Seon Woo Kim, Karen Liljebjelke, Jean Guard, Jo Ann S. Van Kessel

**Affiliations:** a United States Department of Agriculture, Environmental Microbial and Food Safety Laboratory, Beltsville Area Research Center, Agricultural Research Services, Beltsville, Maryland, USA; b United States Department of Agriculture, Russell Research Center, Agricultural Research Services, Athens, Georgia, USA; c United States Department of Agriculture, United States National Poultry Research Center, Athens, Georgia, USA

## Abstract

We report here the draft genome sequences of two *Salmonella enterica* serovar Kentucky eBurstGroup 15 isolates collected from poultry carcasses in Georgia (USA).

## GENOME ANNOUNCEMENT

*Salmonella enterica* serovar Kentucky is a polyphyletic serovar composed of multiple sequence types (STs) within the *S. enterica* subclade A1 lineage that has been associated with poultry and dairy cows in the United States (ST152), poultry and human clinical cases in Europe, Africa, the Middle East, and Asia (ST198), and reptiles and human infections in eastern Europe (ST314 and ST198) (1–8). *S*. Kentucky ST152 is the most frequently isolated serovar/ST from broilers in the United States; however, the mechanisms of persistence in poultry and other animals, such as dairy cows, remain unknown. As part of an ongoing research effort to understand the genomic features involved in the success of *S*. Kentucky eBurstGroup 15 in animals of agricultural significance in the United States, we sequenced the genomes of two isolates collected from poultry carcasses in Georgia.

Isolates were purified on LB agar and subsequently grown overnight at 37°C in LB broth. DNA was extracted using a Qiagen DNeasy kit (Qiagen, Valencia, CA). Sequencing libraries were constructed using a Nextera XT library prep kit (Illumina, La Jolla, CA). Paired-end libraries were loaded onto a NextSeq 500 sequencing platform (Illumina), and the genomes were sequenced using a 2 × 150 approach. Reads were cleaned of contaminants using DeconSeq (9) and Trimmomatic (10) and then assembled using SPAdes version 3.6.2 (11). Phylogenetic inference was conducted using the methods described by Haley et al. (7). Antibiotic resistance genes were identified *in silico* using the ResFinder program (12). Both isolates were evaluated for susceptibility to 25 antimicrobials using an automated microdilution procedure (Sensititre; Thermo Fisher, Lenexa, KS) with specialty plates CVM3AGNF and ESB1F. Antimicrobial MICs were interpreted based upon the epidemiological cutoff values used by the National Antimicrobial Resistance Monitoring System.

Isolate S415 was typed as ST152, while isolate 176.3 has a single nucleotide polymorphism in the *aroC* gene but shared 100% similarity with the other six ST152 alleles. The inclusion of both isolates in the *S*. Kentucky phylogenetic analysis of Haley et al. (7) indicated that 176.3 is a member of the *S*. Kentucky ST152 cluster 2.4.2, and S415 is a member of the 2.4.1.1 cluster. No plasmid sequences were detected in S415, which is atypical of *S*. Kentucky ST152 isolated from poultry in the United States (7, 13). IncI1 and IncX1 plasmid replicons were detected in 176.3.

Isolate S415 was susceptible to all antibiotics, while 176.3 displayed resistance to gentamicin (aminoglycoside) and sulfisoxazole (sulfonamide). In the 176.3 genome, resistance genes *aac(3)-VIa*, *aadA1*, and *sul1* were detected on a contig with sequence similarity to the IncI1 plasmid pCVM29188_101 of *S*. Kentucky CVM29188.

After annotation with the NCBI Prokaryotic Genome Annotation Pipeline (PGAP), the genome sequence of 176.3 encoded a predicted total of 4,722 genes, 21 rRNAs, and 67 tRNAs. The genome sequence of S415 encoded a predicted total of 4,560 genes, 19 rRNAs, and 68 tRNAs.

### Accession number(s).

This whole-genome shotgun project has been deposited at DDBJ/ENA/GenBank under the accession numbers MDTI00000000 and MDTJ00000000. The versions described in this paper are versions MDTI00000000.1 and MDTJ00000000.1, respectively.
